# Bayesian mixture regression analysis for regulation of Pluripotency in ES cells

**DOI:** 10.1186/s12859-019-3331-2

**Published:** 2020-01-02

**Authors:** Mehran Aflakparast, Geert Geeven, Mathisca C.M. de Gunst

**Affiliations:** 1 Department of Mathematics, Vrije Universiteit Amsterdam, De Boelelaan 1081a, Amsterdam, 1081 HV The Netherlands; 2 Hubrecht Institute-KNAW, University Medical Centre Utrecht, Uppsalalaan 8, Utrecht, 3584CT The Netherlands

**Keywords:** Transcription regulation, Pluripotency, Mixture regression, Data integration, Bayesian analysis

## Abstract

**Background:**

Observed levels of gene expression strongly depend on both activity of DNA binding transcription factors (TFs) and chromatin state through different histone modifications (HMs). In order to recover the functional relationship between local chromatin state, TF binding and observed levels of gene expression, regression methods have proven to be useful tools. They have been successfully applied to predict mRNA levels from genome-wide experimental data and they provide insight into context-dependent gene regulatory mechanisms. However, heterogeneity arising from gene-set specific regulatory interactions is often overlooked.

**Results:**

We show that regression models that predict gene expression by using experimentally derived ChIP-seq profiles of TFs can be significantly improved by mixture modelling. In order to find biologically relevant gene clusters, we employ a Bayesian allocation procedure which allows us to integrate additional biological information such as three-dimensional nuclear organization of chromosomes and gene function. The data integration procedure involves transforming the additional data into gene similarity values. We propose a generic similarity measure that is especially suitable for situations where the additional data are of both continuous and discrete type, and compare its performance with similar measures in the context of mixture modelling.

**Conclusions:**

We applied the proposed method on a data from mouse embryonic stem cells (ESC). We find that including additional data results in mixture components that exhibit biologically meaningful gene clusters, and provides valuable insight into the heterogeneity of the regulatory interactions.

## Background

Cell-type- and condition-specific interactions between DNA binding transcription factors (TFs) and their target genes, and modification of chromatin-associated proteins are two primary molecular mechanisms that influence rates of transcription [1, 2]. Uncovering the complex network of regulatory interactions that control spatio-temporal levels of gene expression is crucial for understanding the coordination of biological processes that take place in cells. An important first step is to obtain accurate experimental data. Chromatin immunoprecipitation combined with massively parallel DNA sequencing (ChIP-seq) is now routinely used to determine TF-DNA interactions and genome-wide patterns of histone modifications (HMs) at high resolution. The next step is to infer the relationships between the relevant variables by building plausible and interpretable models. In this paper we propose to infer the functional relationship between gene expression levels and TF binding as well as local chromatin state, via a Bayesian mixture regression analysis that takes into account i) heterogeneity arising from gene-set specific regulatory interaction, ii) the integration of additional biological information such as 3D chromatin interactions or gene function. For this integration the additional data that provide the biological information, need to be transformed into a similarity measure. We study several similarity measures and illustrate the merits of our method to data from mouse embryonic stem cells (ESC).

In a pioneering work [3], Bussemaker and co-authors demonstrated the effectiveness of regression models to study the effect of in silico predicted TF binding on observed gene expression of potential target genes. They proposed multiple linear regression models where discrete counts of TF motifs occurring in gene promoter sequences are used as predictors. Their models describe gene expression as a function of many predictors simultaneously and have been successfully applied to discover binding sites of yeast TFs controlling cell cycle gene expression. Extending this approach to model mammalian gene expression has not been as successful. Complicating factors include the much less compact genomes in higher organisms which include vast stretches of non-coding DNA within and around genes that are rich in regulatory elements and that enable more complex and dynamic regulatory mechanisms. Consequently, the presence of any particular motif in a non-coding region near a gene of interest is a poor predictor of occupancy by a DNA binding protein under a given condition, let alone of any functional relationship. However, several studies have shown that experimentally derived genome-wide ChIP-seq profiles of transcription factors and also HMs, such as H3K4me3, which often marks regions of active transcription, and H3K27me3, a mark of transcriptionally silent chromatin, do correlate strongly with observed levels of gene expression. For instance, Ouyang et al. [4] show that by using a PCA-regression model that includes profiles of 12 different mouse TFs, roughly 65% of gene expression in embryonic stem cells can be explained. This work was extended by integrating HMs and DNA methylation profiles in a later study by Park and Nakai [5]. Karlić et al. [6] employed a regression model to show that patterns of histone modifications are predictive for gene expression. These approaches are similar in the sense that they are built on the premise that a given regulatory signal exerts a universal regulatory effect on all its target genes.

An important point of attention is that individual levels of gene expression do not depend in a simple way on the activity of a single transcription factor. Combinatorial action of multiple transcription factors, local chromatin state and other mechanisms of regulation result in more complex modes of regulation and the contribution of each single transcription factor may therefore be different for different sets of genes. Ordinary least squares (OLS) models are not suitable to identify such divergent effects of regulatory signals, as their output is computed as a single linear combination of regulatory predictors. Ouyang et al. [4] addressed this issue by applying a principle component regression (PCR) analysis which instead of original regulatory signals uses the orthogonal principle component vectors as new predictor variables. Costa et al. [7] took a different approach by fitting mixture regression models, which inherently cluster the data and allow the same regulatory elements to assert different effects on gene expression in distinct clusters of genes. Later, do Rego et al. [8] considered possible correlation of the regulatory elements and used a variable selection approach to detect the most important regulatory signals within each cluster. In spite of the ability of this method to capture data heterogeneity, efficient algorithms are required to optimize the number of mixture components and the tuning parameters. Most importantly, the approach does not accommodate integration of additional data into the model.

Apart from data on genome-wide presence of HMs and TFs, due to recent advances in molecular techniques such as chromosome conformation capture (3C based Hi-C), additional relevant information on a genome-wide scale can be obtained that can be leveraged in a flexible data modelling approach. Hi-C is a proximity-ligation based assay that allows quantification of contact frequencies between pairs of loci genome-wide and as such yields three-dimensional (3D) data on spatial organization and interaction of genes and regulatory elements, that is believed to provide important insight into complex relationships between 3D chromatin structure and gene activity [9]. Another example is functional annotation of genes, that is often used to evaluate clustering methods from a biological perspective, but can also be used as an auxiliary attribute to increase the accuracy of data modelling.

In this study we approach the problem by mixture modeling with a flexible Bayesian estimation procedure. In addition to enabling variable selection, our method gives proper account to incorporation of prior biological knowledge from different, but related, data sources with the aim of enhancing clustering accuracy. The auxiliary gene attributes in this approach are translated into a single similarity matrix which is then incorporated into the prior for (conditional) component membership. Kirk et al. [10] proposed a similar data integration method that works by fitting separate mixture models for each additional data set and linking them through a conditional prior on the mixture component memberships. Similar to their approach we are interested in integration of additional data at the level of component memberships. However, firstly, instead of many we fit only one mixture model and, secondly, we work in a *regression* setting which allows for modelling the dependency of an independent variable of interest on multiple independent variables. Here, we further compare several functions that measure similarity of gene pairs based on various auxiliary attributes and study their influence on the accuracy of our models.

## Results

### External data and similarity measure selection

**Fig. 1:**
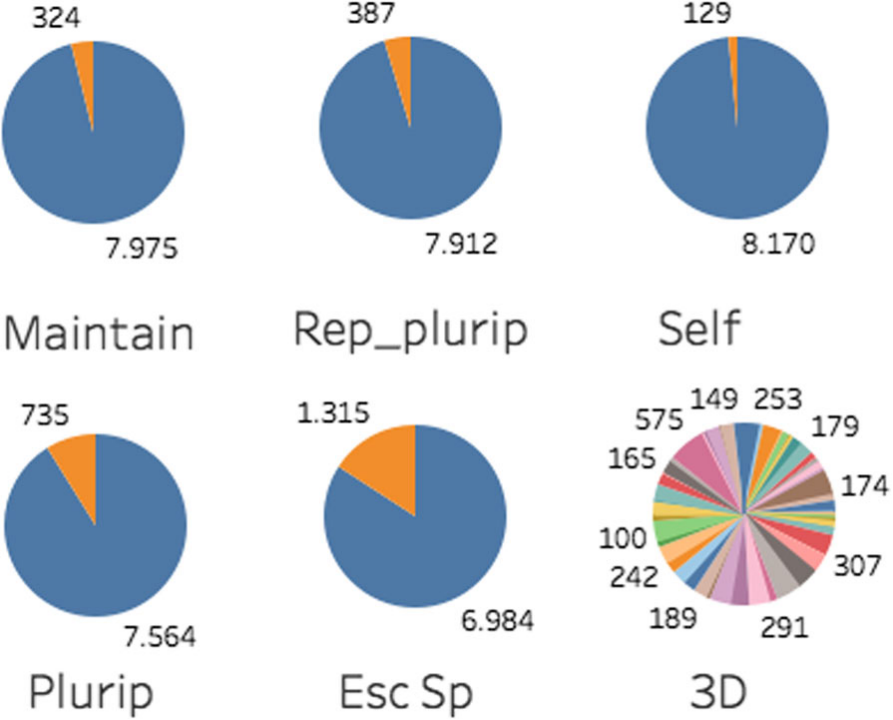
Performance of different similarity measures for the additional data sources. The bars present the MSE of the mixture models that were fitted with DIMR using different similarity matrices that were obtained from the same auxiliary attributes

We first determined which set of additional data can help predicting gene expression levels the best. This was done by a search over different similarity matrices that were obtained from one single or from a combination of additional data (i.e. the auxiliary attributes). The similarity values were calculated using the three functions (7), (10), (12) that were introduced in “Similarity measures” section. The resulting similarity matrices were separately plugged in into our DIMR algorithm. To quantify the differences between the resulting models, the mean squared errors (MSEs) between observed and predicted gene expression values were computed (see “Model evaluation” section for details). We first considered the auxiliary variables separately, as the only source of the additional information about similarity. Figure 1 illustrates the performance of the three similarity functions. In most cases the DIMR-S measure performed far out best, while the performance of Gower’s and Wilson’s measures was comparable; over all conditions DIMR-S performed best.

Having established that DIMR-S yield better predictions in comparison to the other two similarity functions, we continued with the rest of the analysis using DIMR-S. Apart from the eight models with one of the auxiliary variables separately as the additional data set, we considered two models in which the calculation of the similarity values was based on a combination of auxiliary attributes. The first model included a combination of the auxiliary attributes that concerns clustering attributes that represent specific gene functions (Maintain, Plurip, Repplurip, Self, Esc_sp (Set1), while the second model included the combination of all eight auxiliary attributes (Set2).

We further validated the informativeness of the auxiliary attribute sets by comparing the results of the corresponding models with those of a model, denoted by Null, in which all similarity values were set to zero. More importantly, for the last model that we considered, we drew random samples (size=50) from all attribute values and constructed random similarity matrices (Set3).

For the in total twelve models, we utilized the similarities in the DIMR algorithm according to (3)–(5), performed the mixture regression analysis as described in “Mouse embryonic stem cell expression” section, and computed the MSE as indicated in Model validation. To illustrate the benefit of mixture regression, ordinary least squares (OLS) regression was also performed.

**Fig. 2:**
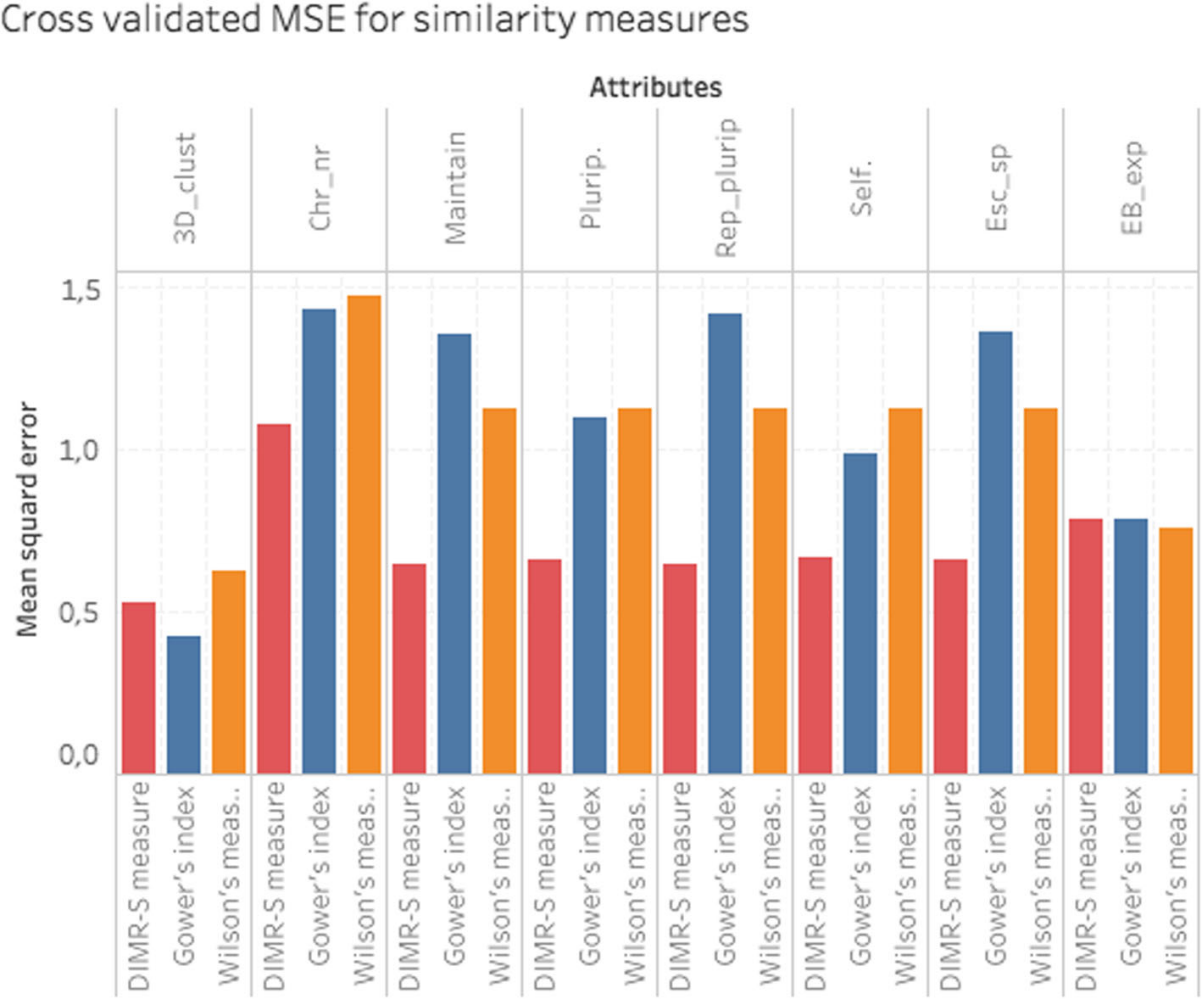
Performance of DIMR using different sources of additional data. The DIMR-S measure is used to calculate similarities, and the cross validated MSEs are presented in ascending order. Set1 and Set2 represent the combination of the functional gene attributes and the combination of all auxiliary attributes, respectively. The results are compared with the mean of the MSE of 50 models whose additional data (Set3) are 50 random surrogates of Set2. The lower and upper values of the error bar for the randomized Set3 represent the first and third quantiles of the resulting MSEs

As depicted in Fig. 2, except for Chr_nr, all auxiliary variables separately yield improved predictions, with similar MSEs, compared to the random model Set3 and the NULL model. However, the MSE drops significantly (t-test, p-value <0.05) when the gene function attributes are combined (Set1); the best result was obtained when all external attributes were combined (Set2). The randomized additional data (Set3) does not show impressive improvement in predictions, as was expected. OLS showed relatively poor performance.

Since the component-wise mixture parameters were estimated in the same manner, the improved results when all auxiliary attributes are used, is due to the clustering stage of the underlying mixture model. As was illustrated by Aflakparast and de Gunst [11], whenever the additional data set is more informative, a better mixture clustering can be expected. Our results show that each additional source of information when utilized individually, has a positive effect ion the predictions. The reason why this effect becomes larger, especially when all additional data are combined, is most likely due to the complementary role of the biological information contained in the auxiliary variables in effective clustering of the genes.

### Mouse embryonic stem cell expression

**Fig. 3:**
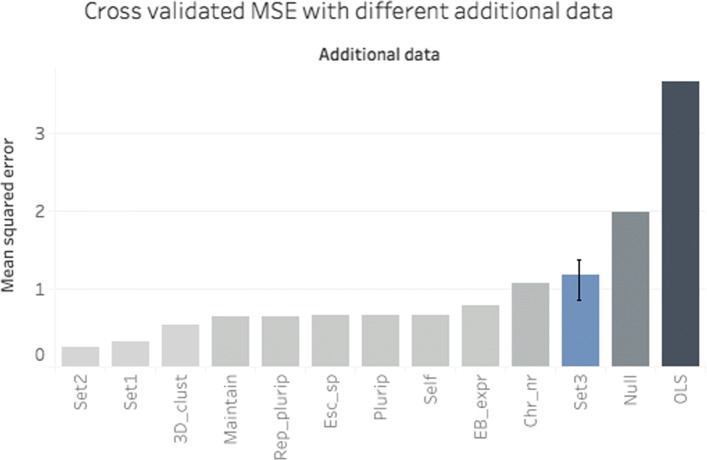
Plots of observed versus predicted mouse ESC gene expression comparing fitted models with DIMR and an OLS methods using different groups of regulatory signals

We now demonstrate that we can recover many crucial regulatory interactions using predictors that represent TF affinities and HM levels derived in silico, allowing us to accurately predict observed gene expression from sequence data. We examined the relationship between gene expression and three groups of regulatory signals TFs, HMs, and a combination of TFs and HMs as explanatory variables. To discover the relationships, we trained three different models using the mixture modelling approach DIMR with a similarity matrix extracted from combining all auxiliary attributes and calculating the similarity of genes using the DIMR-S measure. In addition, we fitted an ordinary multiple linear regression model for comparison. See Fig. 3. The results were compared through 5-fold cross-validated prediction errors. Comparing the proportion of variation of the gene expression levels that has been explained by the models, i.e. the determination coefficient *R*^2^, a slight increase can be seen when both groups of predictors (TFs+HMs) are used as compared to only TFs or only HMs as predictors. However, this increase is reasonably large (13% to 15%) for the DIMR method in relation to that of OLS (4% to 8%). Note that compared to the OLS regression models), the reduction in prediction error for DIMR is considerable (*R*^2^=0.91).

**Fig. 4:**
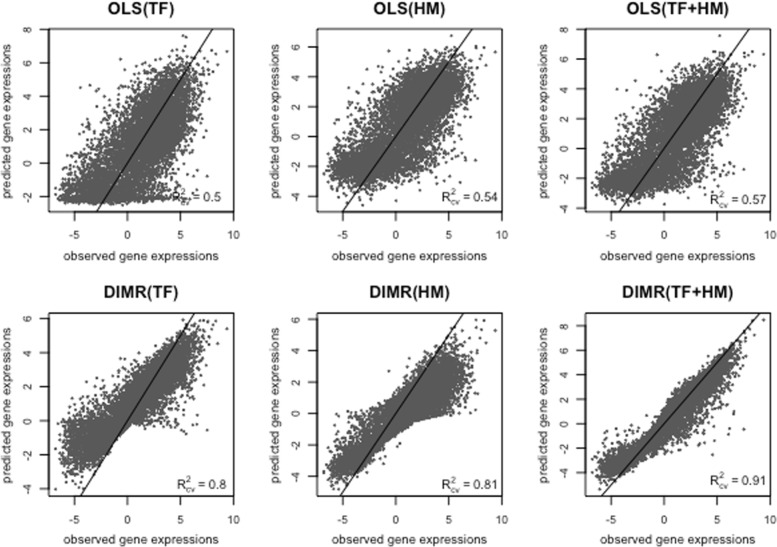
Barplot representing prediction MSEs corresponding to cv-folds of the final model

After it was established that the mixture model with both TFs and HMHs as predictors resulted in the smallest prediction errors, we investigated the results of this model. We first checked whether the final model presented any signals of over-fitting. To this end, we considered the average MSEs corresponding to cross-validated folds separately. As shown in Fig. 4, the prediction errors corresponding to the cv-folds are more or less on the same level, concentrating around 0.2.

**Fig. 5:**
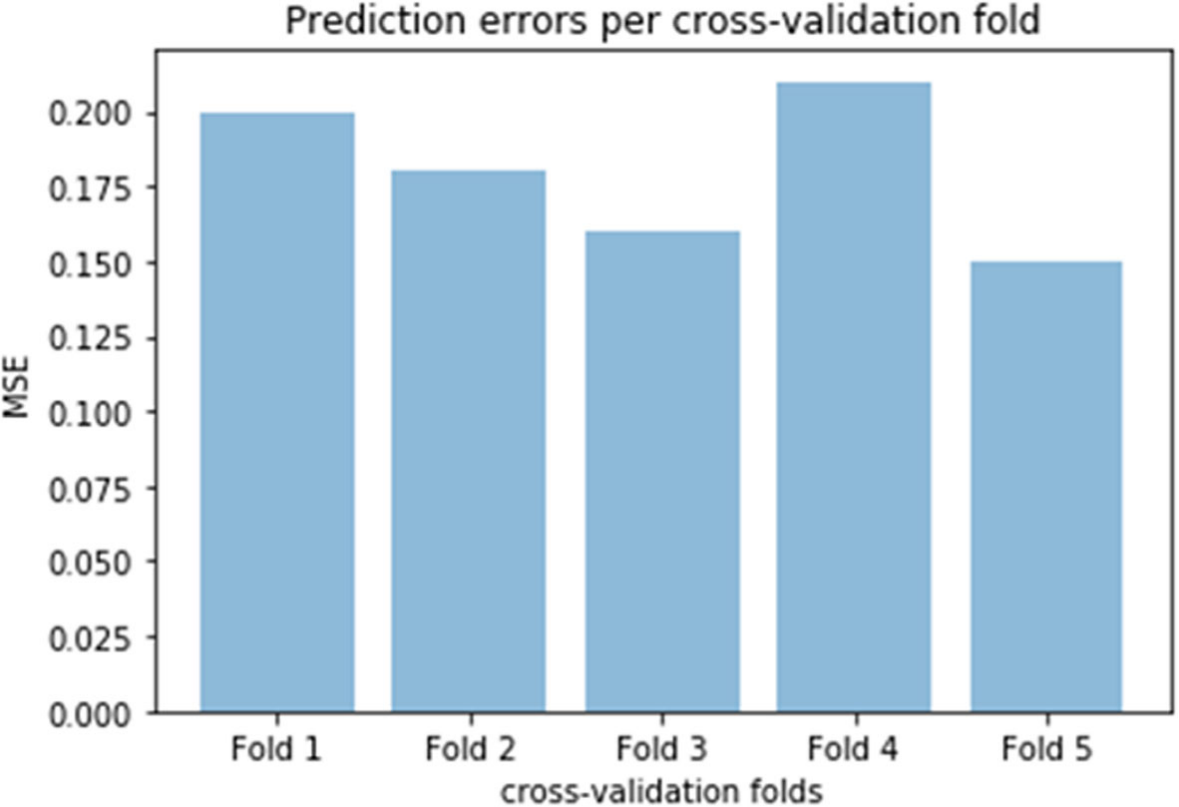
Boxplots for mouse ESC gene expression levels clustered based on mixture modelling using DIMR. The estimated mixture probabilities display the proportion of genes corresponding to each component

The resulting model encompasses the genes in five components, with the number of components being automatically estimated. Figure 5 displays boxplots of the observed gene expression levels for the five estimated mixture components along with the estimated mixture proportions. Comparing the boxplots, we can see different expression behaviour between the genes of different components, especially for the last two components (ANOVA test: *F*(4,8294)=165.1,p-value<2e−16).

**Fig. 6:**
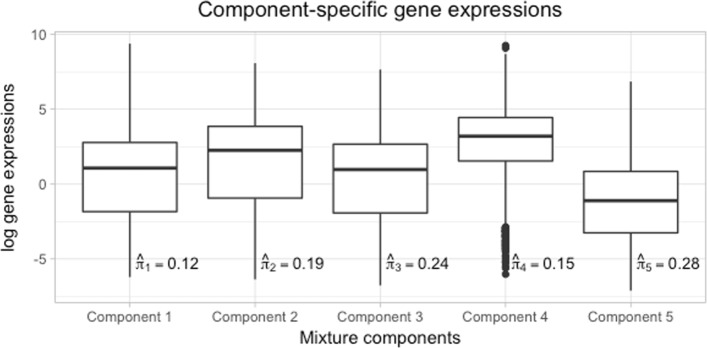
Plots of predicted versus observed mouse ESC gene expression for the 5-component mixture model fitted with DIMR

Observed versus predicted gene expression levels for the 5-component mixture model are depicted in Fig. 6. Notice the distinctive behaviour of component 4 with the smallest prediction accuracy as compared to the other components which can be due to a number of genes with very small expression levels assigned to this component (compare with the boxplot corresponding to component 4 in Fig. 5). This may indicate that this cluster of genes is still somewhat inhomogeneous.

**Fig. 7:**
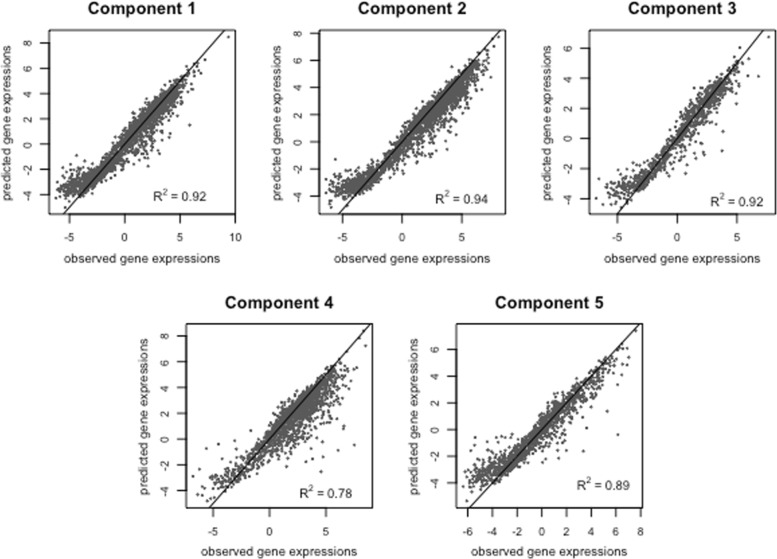
Barplots of regression coefficients and 95% Bayesian credible intervals accros the components of the mixture model fitted with DIMLR on mouse ESC gene expression data

**Fig. 8:**
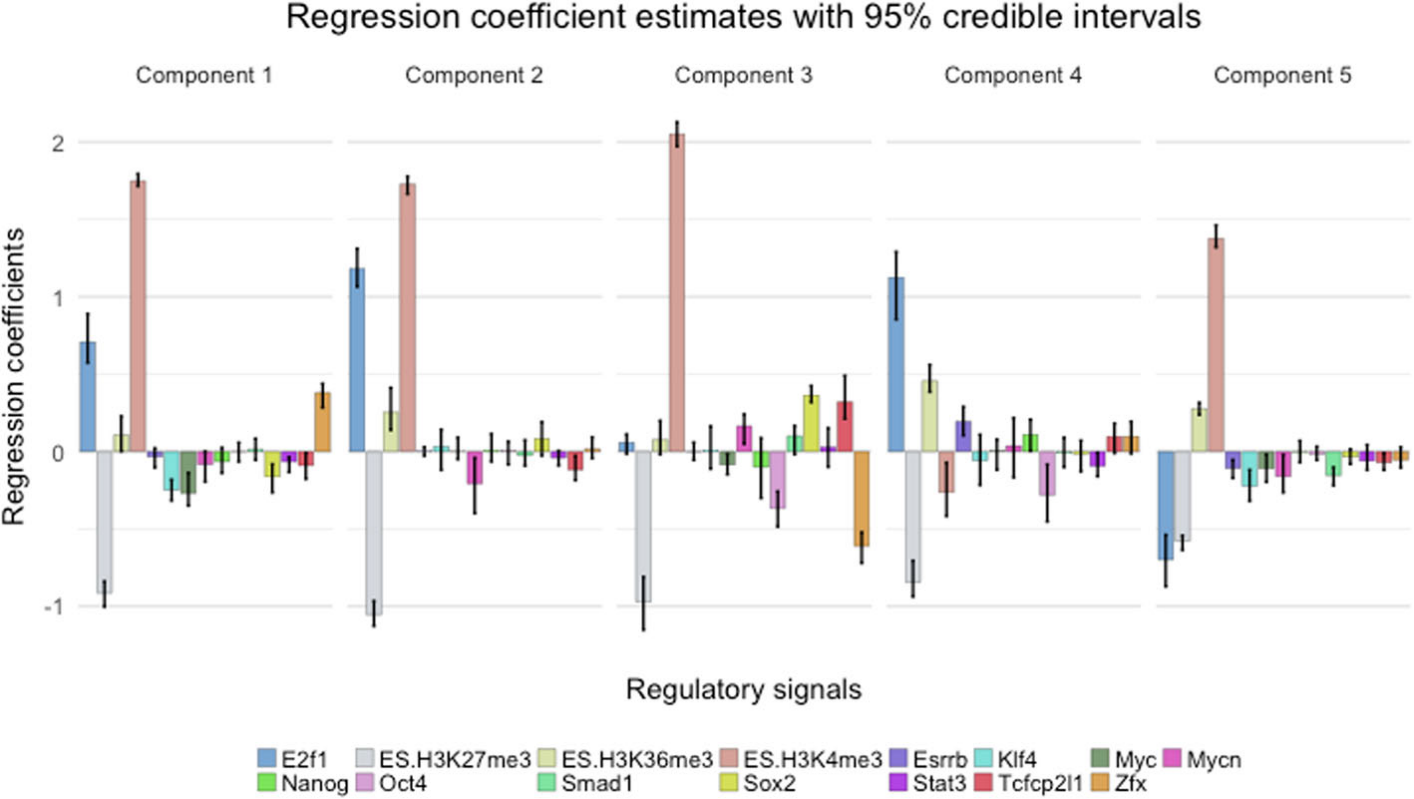
Heatmap of sparsified regression coefficients across the components

We further investigated the regression coefficients to see how the regulatory signals explain gene expression levels across the mixture components. Figure 7 displays the estimated regression coefficients and their corresponding 95% Bayesian credible intervals per component. The credible intervals are then used to sparsify the estimated regression coefficients per component to select the fraction of regulatory signals for which the effects are significantly different from zero. A heatmap of the sparsified regression coefficients is presented in Fig. 8.

Note the overall strong positive effect of the transcriptional regulator E2f1. In a model with only transcriptional regulators, Ouyang et al. [4] found a positive coefficient for E2f1 that dominated the other coefficients, using their PC-regression model. Although we also see mostly positive estimates, it is remarkable that in component 5, which consists of lowly expressed genes, the estimated effect is negative. We also observe that the consistently strong and positive correlation of H3K4me3 and the negative correlation of H3K27me3 are in agreement with findings by others [12–14]. Some signals while being effective in regulation of one group of genes, are demonstrated to play no significant role in regulation in other group of genes. For instance, being consistent with other studies (see, for example, [15] and the references therein) we observe the negative contribution of Oct4 in components 3 and 4, whereas for the rest of the genes no such contribution is present. Perhaps most interesting are the coefficient estimates of the crucial regulators of ESC pluripotency such as Sox2 and Zfx [16]. These factors being significant only in component 1 and 3 (see Fig. 8), surprisingly show completely opposite regulatory effects which was not found in previous studies.

From a biological perspective, we would expect that the clustering into 5 components resulting from our model, would lead to functionally more homogeneous sets of genes, since they should be close together in "expression-regulator" space. To test this hypothesis, we looked at functional enrichment using an online gene set enrichment platform: Enrichr [17]. We particularly focused on GO biological process and GO molecular function terms belonging to GOslim. In each of the components, the genes in all gene sets were tested for significant enrichment using Fisher’s test and a conservative Bonferroni correction was used to correct for multiple testing. The results are shown in the tables provided in the Additional file 1.

## Discussion

Efficient reprogramming of somatic cells to pluripotent cells and subsequent directed differentiation into a lineage of choice holds great potential for regenerative medicine and treatment of human disease. It requires a detailed understanding of the function of the crucial Yamanaka factors Oct4, Sox2, Klf4 and Myc, among others. Identification of their (direct) targets, both in embryonic stem cells and during reprogramming and in iPS cells, remains challenging. Methods that are capable of describing the quantitative regulatory effect of a TF on the activity of a gene in a cell under a certain condition can provide insight into TF-target gene relationships. Given the complex nature of gene regulation in mammals, the different mechanisms of regulation and the multitude of factors involved, methods are needed that can identify and group together genes that share regulatory interactions by integrating different sources of relevant experimental data. It was shown by Ouyang et al. [4] that roughly 65% of the variation in RNA-Seq measured ESC gene expression can be explained using a PC-regression model where ChIP-Seq derived TF affinity scores of 12 TFs are used as predictors. Here, we extended the analysis of ESC gene expression in order to incorporate additional relevant data sets into the model in the following way. To account for differences in chromatin context, which is a reflection of, but also influences, transcriptional activity of a particular locus, we included histone modifications (HMs) as predictors. We used a novel Bayesian mixture approach that simultaneously clusters the genes and fits cluster-specific regression models [11], effectively taking into account the heterogeneity observed when fitting a single model for the entire set of genes. Furthermore, we investigated several similarity functions for extracting information from other types of experiments, most notably chromatin conformation capture assays, which give 3D spatial information. This allows genes to be clustered that share 3D regulatory interactions in addition to the more standard gene functional clusterings often based on Gene Ontology. We compared the different strategies through extensive data analysis.

We showed that our approach explains 91% of the variation in the gene expression levels, as compared to 61% of simple OLS models that were used in previous approaches. We also found that incorporation of both TFs and HMs as predictors results in additional accuracy when compared to models with either set of predictors alone. This is in agreement with the cooperative role of HMs and TFs in determining expression levels [18, 19]. We next showed that including additional relevant biological data in the model resulted in higher predictive accuracy than compared to a model based solely on random additional data, or no additional data at all. Particularly, when combining all additional data the resulting model achieved surprisingly high prediction accuracy, which indicates the substantial benefit of complementary information for the clustering stage of our mixture modelling

We found 5 clusters of genes that are all well explained (component-wise *R*^2^ ranging from 0.81 to 0.94) with distinctive characteristics, which suggests that the clusters are likely to be under the control of different regulatory mechanisms. A challenging but very interesting direction of future work may be to develop rigorous techniques to investigate the dynamic effects of regulatory signals on the changes of gene expressions over time or in different states of related systems. For instance, trying to identify both shared characteristics and differences between embryonic stem cells and induced pluripotent cells from different founder lineages may provide important insights into the regulatory mechanisms that underlie reprogramming and differentiation.

## Conclusion

The proposed method is shown to have higher accuracy compare to the state-of-art methods, and suffer less from prediction errors. The advantage to facilitate data integration makes this method applicable in a wide range of domains where modelling of the main data can be supported by the numerous related additional data sources. Our application on the mouse ESC data results in new insights regarding genetic regulations. Our method identifies clusters which are more homogeneous in terms of their TF regulation and identifies cluster specific variable importance for crucial transcriptional regulators. Here, this allows us to start to disentangle different cellular ES phenotypes, such as cell cycle regulation (for which activity of E2F is crucial) and pluripotency, which is known to be controlled by Sox2, Oct4, KLF4 and c-Myc.

## Methods

### Data

#### Main data

The results in Ouyang et al. [4] suggest that microarrays are less sensitive to detect lowly expressed genes than deep RNA-Seq. Therefore, we use gene expression data measured by RNA-Seq in mouse embryonic stem cells (ESC) as reported in [4]. The expression levels were calculated according to the RPKM definition [20]. These data are available online at http://www.pnas.org/content/106/51/21521?tab=ds. In a regression setting, they are represented by the response variable. On the expression levels of the 15512 genes we examined the regulatory effects of 15 genetic markers (12 TFs and 3 HMs).

As primary set of regulatory predictors we consider the ChIP-Seq data of 12 mouse TFs in ESCs namely E2f1, Mycn, Zfx, Myc, Klf4, Tcfcp2l1, Esrrb, Nanog, Oct4, Sox2, Stat3, Smad1, which were downloaded from NCBI GEO under the accession designation GSE11431 [21]. Mikkelson et al. published genome-wide maps of chromatin state in mouse ESCs based on ChIP-Sequencing of several HMs [22]. From GEO, we obtained the densities (at 25 bp resolution) of H3K4me3, H3K27me3 and H3K36me3 and mapped these to promoter regions (2000 bp up- and down-stream of TSS) of RefSeq genes. In a regression setting we refer to the TF and HM data as explanatory variables or predictors. Whereas Ouyang et al. [4] applied regression on PCA variables, our approach uses the ChIP-Seq signals in the regression model directly. The reason for this lies in the fact that the number of regulatory markers was not too large and PCA analysis often suffers from interpretability issues. Moreover, instead of a separate feature selection step, the presented approach enables shrinkage estimation, where the estimated regression coefficients of less important features automatically tend to be close to zero.

#### Additional data

Throughout this article the following data are used as additional data to improve the accuracy of the model that we use to explain gene-TF or gene-HM relationships. We use eight data sources in the form of auxiliary variables. These data include i) a set of 3D chromatin interactions from Capture-Hi-C linking promoters of mouse genes based on 3D proximity, denoted by 3D_clust [23]. ii) five Boolean attributes, four of which represent the gene functions maintenance (Maintain), pluripotency (Plurip), repression of pluripotency (Repplurip), self renewal (Self), and one, called Esc_sp, which indicates whether or not a gene is critical to specific mouse embryonic stem cell phenotypes, i.e. "mouse ES cell specific genes", iii) gene expression RNA-Seq profiles of embryoid body (EB) [4], denoted by EB_exp, and iv) gene chromosome numbers denoted by Chr_nr.

### Mixture regression model

Let the response vector **Y**=(*Y*_1_,…,*Y*_*n*_)^*T*^ be the *n*-vector that represents the (log) gene expression for a set of *n* genes, *X*_*i*_ the *p*-vector containing the values of *p* explanatory variables for the *i*-th gene, and **X**=(*X*_1_,…,*X*_*n*_)^*T*^ the *n*×*p* -matrix of all explanatory values. We will always work conditionally on *X*_*i*_=*x*_*i*_. We assume that the set of response variables comprises *K* unknown clusters (or components), that *Y*_1_|*x*_1_,…,*Y*_*n*_|*x*_*n*_ are independent, and that *Y*_*i*_|*x*_*i*_ follows a finite mixture of Gaussians given by

1 $$ Y_{i}| x_{i}, \boldsymbol{\beta}, \boldsymbol{\sigma}^{2}, \boldsymbol{\pi} \sim \sum^{K}_{k=1} \pi_{k} \mathcal{N}\left(x_{i}^{T}\beta_{k}, \sigma^{2}_{k}\right), \quad i=1,\ldots,n,   $$

where

$${\begin{aligned} \boldsymbol{\beta}&=(\beta_{1},\ldots,\beta_{K})^{T}, \quad \beta_{k}=(\beta_{k1},\ldots,\beta_{{kp}})^{T},\\ \quad \boldsymbol{\sigma}^{2}&=\left(\sigma^{2}_{1},\ldots,\sigma^{2}_{K}\right)^{T}, \quad \boldsymbol{\pi}=(\pi_{1},\ldots,\pi_{K})^{T}, \end{aligned}} $$

$\sum ^{K}_{k=1}\pi _{k}=1$, and $\mathcal {N}(x_{i}^{T}\beta _{k}, \sigma ^{2}_{k})$ stands for the univariate normal distribution with mean $x_{i}^{T}\beta _{k}$ and variance $\sigma ^{2}_{k}$. This means that *β*_*k*_ denotes the *p*-vector of regression coefficients corresponding to component *k*, *k*=1,…,*K*. The number of components *K* is assumed to be unknown.

Throughout the paper we use capital letters to denote random variables, random vectors or random matrices, and small letters for their realizations; bold type is used for quantities belonging to the full model, and subscript or superscript *k* for quantities belonging to the *k*th cluster.

To estimate the unknown values of the parameters ***β***,***σ***^2^,***π***, and the number of components *K*, we take a Bayesian approach and approximate the posterior distributions by means of the DIMR algorithm that was proposed in [11]. Estimation with this algorithm is based on a vector of latent variables **Z**=(*Z*_1_,…,*Z*_*n*_)^*T*^, where *Z*_*i*_ represents the component membership of data point *i*, so that

2 $$ Y_{i}|x_{i}, \mathbf{z},\boldsymbol{\beta},\sigma^{2} \sim \mathcal{N}(x_{i}^{\intercal}\beta_{z_{i}}, \sigma^{2}_{z_{i}}), \quad i=1,\ldots,n.   $$

This means that as soon as component memberships become known, the gene expression level of gene *i* in component *k* is explained by its corresponding vector of predictors *x*_*i*_ through component-specific parameters ***β***_*k*_ and $\boldsymbol { \sigma ^{2}_{k}}$.

One important aspect of DIMR is its ability to leverage additional similarity information about the data points, summarized by an *n*×*n* symmetric similarity matrix **S**, to improve clustering. The similarity values $\phantom {\dot {i}\!}S_{ii^{\prime }}$ in **S** are used to form a non-exchangeable prior distribution for the component memberships

3 $$ {\begin{aligned} sp(Z_{i}=k | z_{-i}, \mathbf{s},\alpha)= \left\{\begin{array}{ll} n_{-i,k}^{\ast}h_{i}(k)/c\,, & \text{if }\, {k} \text{ is an existing component,}\\ \alpha/c\,, & \text{if \,} {k} \text{ is a new component.} \end{array}\right.  \end{aligned}}  $$

Here *z*_−*i*_ is the (*n*−1)-vector obtained from **z** by deleting *z*_*i*_,*α*>0, *c* is a normalizing constant, and

4 $$ n_{-i,k}^{\ast}=\sum_{i':i'\ne i}I_{\{s_{ii'}\geq T_{i}\}}I_{\{z_{i'}=k\}}.  $$

The function *h*_*i*_(*k*) in (3) is of the form

5 $$ h_{i}(k)=1+\sum_{i':i'\ne i} s_{ii'}I_{\{z_{i'}=k\}}   $$

and represents the overall similarity of gene *i* with all other genes in component *k*. In the above, *I* denotes the indicator function, and *T*_*i*_ is a threshold value to ensure that gene *i* is more likely to end up in a component where it has high similarity with the majority of the other genes. In this study *T*_*i*_ is defined as the third quantile of the similarity values of pairs of the genes in the same cluster as gene *i* is assigned to.

Evidently, given the component membership vector **Z=z**, the estimation problem turns into fitting *K* independent regression models. In order to obtain a biologically plausible and interpretable model, we impose additional restrictions on the ***β***_*k*_ vectors by assuming that in each cluster *k* only a few explanatory signals contribute to the variability of the response gene expressions. This is applied by shrinkage estimation of the regression coefficients as in the *Bayesian Lasso* procedure of [24].

Although the Bayesian Lasso shrinks the regression coefficients *β*_*kj*_ towards zero, it does not yield sparse solutions. In order to achieve sparsity, we apply a post-hoc selection analysis via (quantile-based) Bayesian credible intervals. For further details on our mixture modeling procedure we refer the reader to [11].

### Similarity measures

In general, the similarity matrix **S** does not need to be directly available, but, as explained by Aflakparast and de Gunst [11], it can be obtained from a single additional attribute or combination of several auxiliary attributes by means of a similarity measure. In this study, we have access to the eight auxiliary attributes described in “Additional data” section. Here we introduce and compare a set of functions that define such measures so that they are suitable to be used within DIMR.

Since the additional information can appear in the form of categorical or continuous auxiliary attributes, we focus on similarity measures that are applicable for mixed attributes. Numerous similarity (or distance) measures for either categorical or continuous attributes have been proposed and studied in different disciplines (see e.g. the review studies [25] and [26]). However, there is a limited number of studies that consider a mixed data situation. The indexes proposed by Wilson and Martinez [27] and Gower [28] are especially popular in applications. The behaviour of these measures have been tested in various multivariate statistical analysis and data mining contexts ranging from clustering and classification problems to (kernel) principal component analysis, yet not in a mixture model context. In our mixture regression setting, we study specific similarity functions that suit our application. We then assess the ability of these functions, when they are used to produce similarity matrices **S** within our mixture regression model framework.

Similarity functions that only apply to data with categorical attributes can handle mixed data often through discretization of continuous valued attributes which may cause loss of available information, see [29]. A good example is Gower’s index as proposed by Gower [28]. There the similarity $s^{G}_{{ijm}}$ of two data points *i* and *j* measured for attribute *A*^*m*^,*m*=1,…*M* is given by

6 $$ {\begin{aligned} s^{G}_{{ijm}}= \left\{\begin{array}{ll} 1 & \!\!\!\!\!\text{if}\, A^{m} \,\text{is a categorical attribute and} \, A^{m}_{i}\,=\,A^{m}_{j}; \text{or} A^{m}_{i}\,=\,A^{m}_{j}\!=,\\ & \!\!\!0~\text{(when double zeros are included in the comparison)}\\ 0 & \!\!\!\text{if}\, A^{m} \,\text{is a categorical attribute and:} \, A^{m}_{i}\neq A^{m}_{j}\qquad,\\ 1-\frac{|A^{m}_{i}-A^{m}_{j}|}{max(A^{m})-min(A^{m})} & \!\!\text{if} \, A^{m} \,\text{is a continuous-valued attribute}. \end{array}\right. \end{aligned}}  $$

The total similarity between *i* and *j* for *M* attributes is given by

7 $$ \text{Gow-}S_{{ij}}=\frac{\sum_{m=1}^{M} \delta_{{ijm}} s^{G}_{{ijm}}}{\sum_{m=1}^{M} \delta_{{ijm}}}   $$

where *δ*_*ijm*_ equals one, unless one or both values are unknown, or double zeros are excluded from the comparison, in which case *δ*_*ijm*_ equals zero.

Gower’s index is widely used and allows for an efficient treatment of missing data and the inclusion of variable weights. However, a disadvantage is that the similarity of data points for categorical variables is simply an overload measure, which ignores a large amount of information provided by differences in categorical attribute values. Wilson et al. proposed three distance measures in classification contexts where the data come with pre-defined output classes [27]. Their Heterogeneous Value Difference Metric (HVDM) is one of the measures that use the original form of continuous attributes without discretization. HVDM calculates the distance *d*_*ijm*_ between data points *i* and *j* for the *m*-th attribute *A*^*m*^ as

8 $$ {\begin{aligned} d_{{ijm}}= \left\{\begin{array}{ll} 1 & \text{if} \,A^{m}_{i} \, \text{or}\, A^{m}_{j} \,\text{are unknown},\\ {\sum_{c=1}^{C}|\frac{n_{A^{m}_{i},c}}{n_{A_{i}}}-\frac{n_{A^{m}_{j},c}}{n_{A^{m}_{j}}}|} & \text{if}\, A^{m} \, \text{is a categorical attribute},\\ {\frac{|A^{m}_{i}-A^{m}_{j}|}{4\sigma_{A^{m}}}} & \text{if}\, A^{m} \, \text{is a continuous attribute,} \end{array}\right. \end{aligned}}  $$

where $\phantom {\dot {i}\!}\sigma _{A^{m}}$ is the standard deviation of the continuous attribute $A^{m}, n_{A^{m}_{i},c}$ and $n_{A^{m}_{i}}$ are the number of data points in class *c*, and the total number of data points for which $A^{m}=A^{m}_{i}$, respectively.

To be consistent with our study we consider a similarity based version of (8) with $s^{W}_{{ijm}}=1-d_{{ijm}}$ and assume no pre-defined output class information:

9 $$  s^{W}_{{ijm}}= \left\{\begin{array}{ll} 0 & \text{if}\, A_{i} \, \text{or} \, A_{j} \, \text{are unknown},\\ 1-{|\frac{n_{A_{i}}-n_{A_{j}}}{n}|} & \text{if} \, {A} \, \text{is a categorical attribute},\\ 1-{\frac{|A_{i}-A_{j}|}{4\sigma_{A}}} & \text{if}\, {A} \, \text{is continuous attribute}, \end{array}\right.  $$

and the total similarity between *i* and *j* for *M* attributes is

10 $$  \text{Wilson-}S_{{ij}}=\frac{\sum_{m=1}^{M} \delta_{{ijm}} s^{W}_{{ijm}}}{\sum_{m=1}^{M} \delta_{{ijm}}}.  $$

**Fig. 9:**
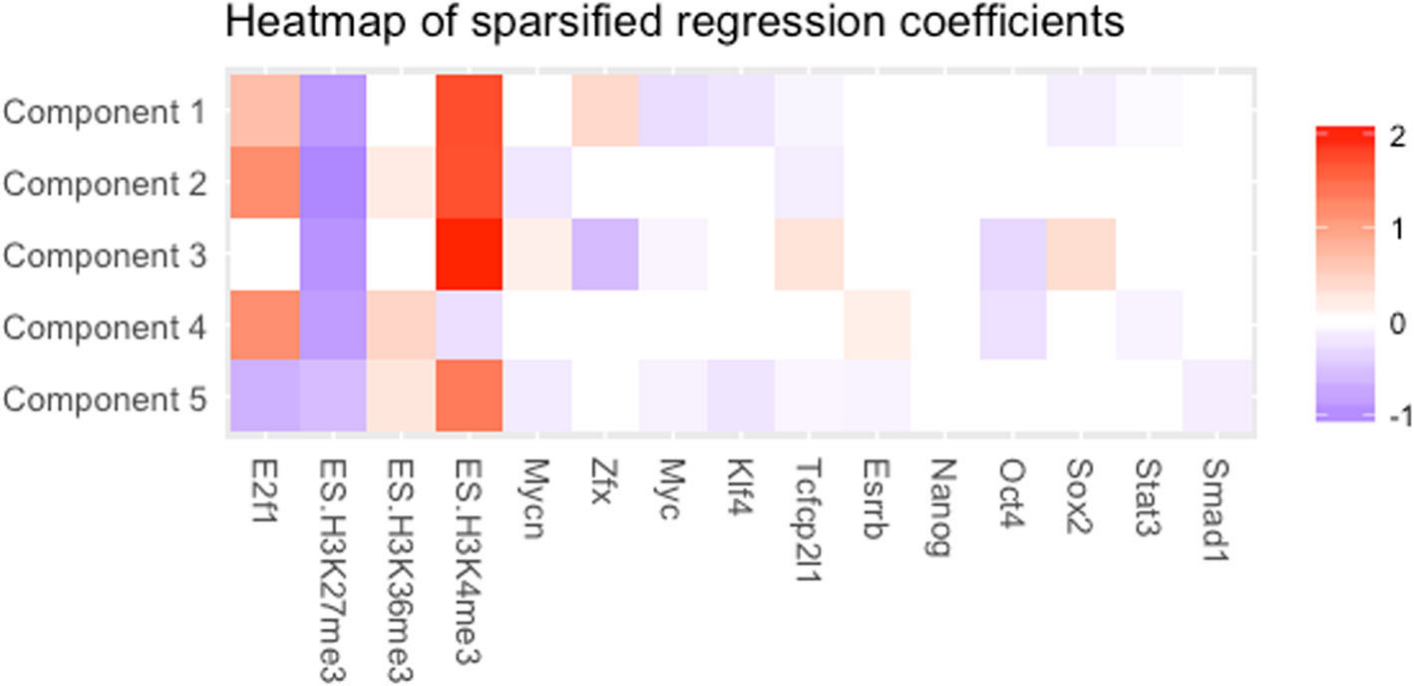
Frequency per class for the auxiliary attributes of categorical type

To see if these measures are suitable for our application the frequency of the attribute values for the seven categorical attributes that are used in this study are presented in Fig. 9. It is evident that for attributes 3–7 there is a big gap between the frequency of the two values. These attributes are Boolean attributes that indicate whether or not a gene belongs to a specific functional class that relates to a specific mouse embryonic stem cell phenotype (i.e. maintenance, pluripotency, repression of pluripotency, self renewal, and general mouse ES cell specific genes). Naturally, a good similarity measure should assign higher similarity to the genes that are classified in the same (rare) functional class (i.e. TRUE–TRUE) as opposed to the case that both are not (FALSE–FALSE). To some extent, this fact is neglected in both Gower’s and Wilson’s measures. Therefore we propose the following similarity measure, $s^{D}_{{ijm}}$ for mixed auxiliary data.

11 $$ {\begin{aligned} s^{D}_{{ijm}}= \left\{\begin{array}{ll} 0 & \text{if} \, A_{i} \,\text{or}\, A_{j} \, \text{is unknown, or if} \, {A} \, \text{is a categorical}~\\& \text{attribute and:} \, A_{i}\neq A_{j},\\ 1-{\frac{n_{A_{i}}(n_{A_{j}}-1)}{n(n-1)}} & \text{if} \, {A} \, \text{is a categorical attribute and} \, A_{i}=A_{j},\\ 1-{\frac{|A_{i}-A_{j}|}{max(A)-min(A)}} & \, \text{if} \, {A} \, \text{is a continuous valued attribute}, \end{array}\right. \end{aligned}}  $$

and the total similarity between *i* and *j* for *M* attributes becomes

12 $$ \text{DIMR-}S_{{ij}}=\frac{\sum_{m=1}^{M} \delta_{{ijm}} s^{D}_{{ijm}}}{\sum_{m=1}^{M} \delta_{{ijm}}}.  $$

We chose the Manhattan metric for continuous attributes, like in Gower’s and Wilson’s metrics. For normalization, we followed Gower’s index as the range is easy to calculate. Note that for a categorical attribute when two data points belong to the same group, rare groups are assigned higher similarity values compared to the frequent ones.

### Model evaluation

We evaluated fitted mixture models for the three similarity measures that were introduced in “Similarity measures” section to investigate which of them exploits best the auxiliary data.

Model evaluation is an important part of the procedure for data integration, especially in this specific application with more than one auxiliary attribute. In principle, the similarity matrix **S** can be calculated in different ways using different similarity functions on different combination of auxiliary attributes. Let $\mathcal {F}$ be the set of functions to measure the similarity of genes, and $\mathcal {A}$ the set of all combination of auxiliary attributes from which **S** can be obtained. The purpose of this procedure is to 1) evaluate the performance of the similarity functions $\mathcal {F}$ and select an optimal one, and 2) employ the selected function to evaluate and choose among models whose additional data are obtained from $\mathcal {A}$. Here this is done by means of *H*-fold cross-validation (CV). The data set is randomly split into *H* approximately equal sized groups. Throughout this study, we use 5-fold cross validation to avoid additional costs of using higher-fold settings. Each group is left out once, while the other groups are used to estimate the model parameters. With the estimates of the parameters at hand, the predicted expression level for gene *i* in the test set *h* is calculated from

13 $$ \hat{y}_{i}=\sum_{k=1}^{K^{(-h)}} p(Z_{i}=k|\,y_{i},\,x_{i},\mathbf{S}_{(f,A)})\, x_{i}^{T} \boldsymbol{\beta}_{k}^{(-h)}  $$

where **S**_(*f,A*)_ is the similarity matrix obtained from the auxiliary attribute set *A* using similarity function *f*, and *K*^(−*h*)^ and $\boldsymbol {\beta }_{k}^{(-h)}$ the estimated number and vector of parameter estimates, respectively, corresponding to component *k* obtained from the training set leaving out the test set *h*. We calculated the mean squared error (MSE) for the test set *h* with *n*^(*h*)^ genes by

14 $$ MSE_{(h)}(f,A)=\frac{1}{n^{(h)}}\sum_{i=1}^{n^{(h)}} (y_{i}-\hat{y}_{i})^{2},   $$

which yielded aggregated cross-validated prediction error as $\tfrac {1}{H}\sum _{h=1}^{H} \,MSE_{(h)}(f,A)$, of which the minimum over *f* and *A* yields the optimal model.

In this study there are situations where we use DIMR without additional data, or where a non-mixture model is applied. In these cases th evaluation of the fitted models is similarly conducted via cross-validation and based on the MSE defined in (14).

## Supplementary information

**Additional file 1** Functional enrichment of the resulting gene clusters.

## Data Availability

The main gene expression data are available online at http://www.pnas.org/content/106/51/21521?tab=ds.

## Abbreviations

3C: Chromosome conformation capture
3D: 3-dimensional
CHIP-seq: Chromatin immunoprecipitation sequencing
Chr-nr: Chromosome number
CV: Cross-validation
DIMR: Data integrative mixture regression
EB-exp: Gene expression RNA-Seq
profiles of embryoid body; EB: Embryonic body
ESC: Embryonic stem cells
GEO: Gene expression omnibus
GO: Gene ontology
HM: Histone modification
HVDM: Heterogeneous value difference metric
Maintain: maintenance
MSE: Mean squared errors
OLS: Ordinary least squares
PCA: Principle component analysis
PCR: Principle component regression
Plurip: Pluripotency
Repplurip: repression of pluripotency
Self: Self renewal
TF: Transcription factors
TSS: Transcription start sites

## References

[1] Goldberg AD, Allis CD, Bernstein E (2007). Epigenetics: a landscape takes shape. Cell.

[2] Bibikova M, Laurent LC, Ren B, Loring JF, Fan JB (2008). Unraveling epigenetic regulation in embryonic stem cells. Cell Stem Cell.

[3] Bussemaker H, Li H, Siggia E (2001). Regulatory element detection using correlation with expression [Article]. Nat Genet.

[4] Ouyang Z, Zhou Q, Wong WH (2009). ChIP-Seq of transcription factors predicts absolute and differential gene expression in embryonic stem cells. Proc Natl Acad Sci.

[5] Park SJ, Nakai K (2011). A regression analysis of gene expression in ES cells reveals two gene classes that are significantly different in epigenetic patterns. BMC Bioinformatics.

[6] Chung HR, Lasserre J, Vlahoviček K, Vingron M, Karlić R (2010). Histone modification levels are predictive for gene expression. Proc Natl Acad Sci.

[7] Costa IG, Roider HG, do Rego TG, de Carvalho FdA (2011). Predicting gene expression in T cell differentiation from histone modifications and transcription factor binding affinities by linear mixture models. BMC Bioinformatics.

[8] do Rego T. G., Roider H. G., de Carvalho F. A. T., Costa I. G. (2012). Inferring epigenetic and transcriptional regulation during blood cell development with a mixture of sparse linear models. Bioinformatics.

[9] Lieberman-Aiden E, Van Berkum NL, Williams L, Imakaev M, Ragoczy T, Telling A (5950). Comprehensive mapping of long-range interactions reveals folding principles of the human genome. Science.

[10] Kirk P, Griffin JE, Savage RS, Ghahramani Z, Wild DL (2012). Bayesian correlated clustering to integrate multiple datasets. Bioinformatics.

[11] Aflakparast M, Gunst M. Data integrative Bayesian inference for mixtures of regression models. J R Stat Soc Ser C (Appl Stat). 2019:03. 10.1111/rssc.12346.

[12] Cao R, Wang L, Wang H, Xia L, Erdjument-Bromage H, Tempst P (2002). Role of histone H3 lysine 27 methylation in Polycomb-group silencing. Science.

[13] van Ingen H, van Schaik FM, Wienk H, Ballering J, Rehmann H, Dechesne AC (2008). Structural insight into the recognition of the H3K4me3 mark by the TFIID subunit TAF3. Structure.

[14] Barski A, Jothi R, Cuddapah S, Cui K, Roh TY, Schones DE (2009). Chromatin poises miRNA-and protein-coding genes for expression. Genome Res.

[15] Liang J, Wan M, Zhang Y, Gu P, Xin H, Jung SY (2008). Nanog and Oct4 associate with unique transcriptional repression complexes in embryonic stem cells. Nat Cell Biol.

[16] Chambers I, Smith A (2004). Self-renewal of teratocarcinoma and embryonic stem cells. Oncogene.

[17] Chen EY, Tan CM, Kou Y, Duan Q, Wang Z, Meirelles GV (2013). Enrichr: interactive and collaborative HTML5 gene list enrichment analysis tool. BMC Bioinformatics.

[18] McLeay RC, Lesluyes T, Cuellar Partida G, Bailey TL (2012). Genome-wide in silico prediction of gene expression. Bioinformatics.

[19] Lawrence M, Daujat S, Schneider R (2016). Lateral thinking: how histone modifications regulate gene expression. Trends Genet.

[20] Mortazavi A, Williams BA, McCue K, Schaeffer L, Wold B (2008). Mapping and quantifying mammalian transcriptomes by RNA-Seq. Nat Methods.

[21] Chen X, Xu H, Yuan P, Fang F, Huss M, Vega VB (2008). Integration of external signaling pathways with the core transcriptional network in embryonic stem cells. Cell.

[22] Mikkelsen Tarjei S., Ku Manching, Jaffe David B., Issac Biju, Lieberman Erez, Giannoukos Georgia, Alvarez Pablo, Brockman William, Kim Tae-Kyung, Koche Richard P., Lee William, Mendenhall Eric, O’Donovan Aisling, Presser Aviva, Russ Carsten, Xie Xiaohui, Meissner Alexander, Wernig Marius, Jaenisch Rudolf, Nusbaum Chad, Lander Eric S., Bernstein Bradley E. (2007). Genome-wide maps of chromatin state in pluripotent and lineage-committed cells. Nature.

[23] Schoenfelder Stefan, Furlan-Magaril Mayra, Mifsud Borbala, Tavares-Cadete Filipe, Sugar Robert, Javierre Biola-Maria, Nagano Takashi, Katsman Yulia, Sakthidevi Moorthy, Wingett Steven W., Dimitrova Emilia, Dimond Andrew, Edelman Lucas B., Elderkin Sarah, Tabbada Kristina, Darbo Elodie, Andrews Simon, Herman Bram, Higgs Andy, LeProust Emily, Osborne Cameron S., Mitchell Jennifer A., Luscombe Nicholas M., Fraser Peter (2015). The pluripotent regulatory circuitry connecting promoters to their long-range interacting elements. Genome Research.

[24] Park T, Casella G (2008). The bayesian lasso. J Am Stat Assoc.

[25] Boriah S, Chandola V, Kumar V. Similarity measures for categorical data: A comparative evaluation. In: Proceedings of the 2008 SIAM International Conference on Data Mining. SIAM: 2008. p. 243–54. 10.1137/1.9781611972788.22.

[26] Cha SH (2007). Comprehensive survey on distance/similarity measures between probability density functions. City.

[27] Wilson DR, Martinez TR (1997). Improved heterogeneous distance functions. J Artif Intell Res.

[28] Gower J. C. (1971). A General Coefficient of Similarity and Some of Its Properties. Biometrics.

[29] McCane B, Albert M (2008). Distance functions for categorical and mixed variables. Pattern Recogn Lett.

