# Supplementary material for: Bayesian mixture regression analysis for regulation of Pluripotency in ES cells
Source: BMC Bioinformatics. 2020 Jan 2;21:3. doi: 10.1186/s12859-019-3331-2 (PMC6941360; doi:10.1186/s12859-019-3331-2)
Supplement: Supplementary file 1 — Additional file 1 Functional enrichment of the resulting gene clusters. [file 12859_2019_3331_MOESM1_ESM.pdf]

Component 1

GO Biological Process 2015

Name	P-value	Adjusted p-value	Combined score
tissue morphogenesis (GO:0048729)	2.177e-7	0.001019	16.39
morphogenesis of an epithelium (GO:0002009)	0.000005273	0.008224	11.28
sensory organ development (GO:0007423)	0.000003747	0.008224	11.05
embryonic morphogenesis (GO:0048598)	0.00001647	0.01926	9.59
radial glial cell differentiation (GO:0060019)	0.00002771	0.02593	8.72
regulation of synaptic transmission (GO:0050804)	0.00004279	0.03337	8.08
synaptic transmission (GO:0007268)	0.00005753	0.03587	7.78
sodium ion transmembrane transport (GO:0035725)	0.00006305	0.03587	7.43
regulation of neural precursor cell proliferation (GO:2000177)	0.00006900	0.03587	7.08
nervous system development (GO:0007399)	0.0001167	0.05462	6.63

GO Molecular Function 2015

Name	P-value	Adjusted p-value	Combined score
sequence-specific DNA binding RNA polymerase II transcription factor activity (GO:0000981)	1.516e-8	0.00001514	27.11
RNA polymerase II regulatory region sequence-specific DNA binding (GO:0000977)	4.840e-8	0.00001888	26.22
RNA polymerase II regulatory region DNA binding (GO:0001012)	8.175e-8	0.00001888	26.15
RNA polymerase II core promoter proximal region sequence-specific DNA binding transcription factor activity (GO:0000982)	9.119e-8	0.00001888	25.75
transcription regulatory region sequence-specific DNA binding (GO:0000976)	1.379e-7	0.00002212	25.56
RNA polymerase II core promoter proximal region sequence-specific DNA binding (GO:0000978)	9.447e-8	0.00001888	25.10
core promoter proximal region sequence-specific DNA binding (GO:0000987)	1.550e-7	0.00002212	24.89
core promoter proximal region DNA binding (GO:0001159)	2.163e-7	0.00002701	24.42
metal ion transmembrane transporter activity (GO:0046873)	0.000003465	0.0003847	19.29
RNA polymerase II core promoter proximal region sequence-specific DNA binding transcription factor activity involved in positive regulation of transcription (GO:0001077)	0.00002306	0.002304	13.26

Component 2

GO Biological Process 2015

Name	P-value	Adjusted p-value	Combined score
regulation of apoptotic signaling pathway (GO:2001233)	4.222e-7	0.0003153	20.43
negative regulation of cellular component organization (GO:0051129)	2.455e-7	0.0003153	20.19
growth (GO:0040007)	7.298e-7	0.0003153	19.33
small molecule biosynthetic process (GO:0044283)	1.982e-7	0.0003153	19.18
hemostasis (GO:0007599)	3.640e-7	0.0003153	19.13
coagulation (GO:0050817)	7.325e-7	0.0003153	19.02
blood coagulation (GO:0007596)	7.325e-7	0.0003153	19.00
organ morphogenesis (GO:0009887)	6.483e-7	0.0003153	18.92
alcohol metabolic process (GO:0006066)	6.006e-7	0.0003153	18.81
mitotic cell cycle (GO:0000278)	5.839e-7	0.0003153	18.48

GO Molecular Function 2015

Name	P-value	Adjusted p-value	Combined score
ATP binding (GO:0005524)	7.126e-7	0.0007390	17.30
chromatin binding (GO:0003682)	0.00003234	0.01677	10.05
protein homodimerization activity (GO:0042803)	0.0001452	0.01882	9.46
sulfur compound binding (GO:1901681)	0.00009432	0.01882	9.20
glycosaminoglycan binding (GO:0005539)	0.0001341	0.01882	9.12
antioxidant activity (GO:0016209)	0.00009545	0.01882	9.00
binding, bridging (GO:0060090)	0.0001241	0.01882	8.82
protein binding, bridging (GO:0030674)	0.0001251	0.01882	8.67
protein C-terminus binding (GO:0008022)	0.0002024	0.02332	8.49
isomerase activity (GO:0016853)	0.0003034	0.03146	7.71

Component 3

GO Biological Process 2015

Name	P-value	Adjusted p-value	Combined score
small GTPase mediated signal transduction (GO:0007264)	0.00001313	0.05176	6.96
regulation of anatomical structure size (GO:0090066)	0.0003149	0.1892	4.06
blood coagulation (GO:0007596)	0.0002950	0.1892	3.95
coagulation (GO:0050817)	0.0002950	0.1892	3.95
tissue morphogenesis (GO:0048729)	0.0003373	0.1892	3.94
hemostasis (GO:0007599)	0.0003838	0.1892	3.93
regulation of endocytosis (GO:0030100)	0.0002654	0.1892	3.86
cell-substrate adhesion (GO:0031589)	0.0002552	0.1892	3.69
positive regulation of transforming growth factor beta receptor signaling pathway (GO:0030511)	0.0007983	0.2421	3.61
brown fat cell differentiation (GO:0050873)	0.0009250	0.2492	3.59

GO Molecular Function 2015

Name	P-value	Adjusted p-value	Combined score
calmodulin binding (GO:0005516)	0.0003942	0.3201	2.75
catecholamine binding (GO:1901338)	0.004353	0.5490	1.78
protein homodimerization activity (GO:0042803)	0.003965	0.5490	1.47
metal ion transmembrane transporter activity (GO:0046873)	0.007393	0.5490	1.47
active transmembrane transporter activity (GO:0022804)	0.007393	0.5490	1.45
guanyl-nucleotide exchange factor activity (GO:0005085)	0.005154	0.5490	1.45
gated channel activity (GO:0022836)	0.007601	0.5490	1.44
Ras GTPase binding (GO:0017016)	0.004365	0.5490	1.43
divalent inorganic cation transmembrane transporter activity (GO:0072509)	0.005086	0.5490	1.41
cadherin binding (GO:0045296)	0.01051	0.5490	1.41

Component 4

GO Biological Process 2015

Name	P-value	Adjusted p-value	Combined score
mitotic cell cycle (GO:0000278)	4.905e-10	0.000002197	30.03
mitotic cell cycle phase transition (GO:0044772)	1.919e-7	0.0004014	18.17
cell cycle phase transition (GO:0044770)	2.688e-7	0.0004014	18.13
negative regulation of transferase activity (GO:0051348)	0.000005489	0.002459	14.54
regulation of protein serine/threonine kinase activity (GO:0071900)	0.000002533	0.002316	14.53
cellular response to hormone stimulus (GO:0032870)	0.00001024	0.002929	14.42
regulation of neuron differentiation (GO:0045664)	0.000004438	0.002316	14.41
negative regulation of cellular component organization (GO:0051129)	0.00001235	0.002929	14.38
negative regulation of protein modification process (GO:0031400)	0.00001047	0.002929	14.23
proteolysis involved in cellular protein catabolic process (GO:0051603)	0.00001242	0.002929	14.17

GO Molecular Function 2015

Name	P-value	Adjusted p-value	Combined score
protein kinase binding (GO:0019901)	7.540e-7	0.0007140	18.49
kinase binding (GO:0019900)	0.000002209	0.001046	17.52
ATPase activity, coupled (GO:0042623)	0.000008997	0.002840	14.76
ATPase activity (GO:0016887)	0.00003306	0.006262	12.81
transcription corepressor activity (GO:0003714)	0.00003098	0.006262	11.79
protein serine/threonine kinase activity (GO:0004674)	0.00005408	0.008536	11.76
transcription factor binding (GO:0008134)	0.00007675	0.01038	10.66
protein kinase regulator activity (GO:0019887)	0.0001058	0.01252	10.18
kinase regulator activity (GO:0019207)	0.0001732	0.01823	9.11
transcription regulatory region sequence-specific DNA binding (GO:0000976)	0.0002489	0.02072	9.00

Component 5

GO Biological Process 2015

Name	P-value	Adjusted p-value	Combined score
cellular metal ion homeostasis (GO:0006875)	0.000002217	0.002224	15.02
cellular chemical homeostasis (GO:0055082)	0.000002632	0.002224	14.94
cellular ion homeostasis (GO:0006873)	0.000004005	0.002418	14.78
behavior (GO:0007610)	9.912e-7	0.002224	14.74
cellular cation homeostasis (GO:0030003)	0.000003496	0.002418	14.73
regulation of muscle contraction (GO:0006937)	0.000002455	0.002224	13.76
cation homeostasis (GO:0055080)	0.000006182	0.003265	13.76
metal ion homeostasis (GO:0055065)	0.000007315	0.003434	13.70
regulation of smooth muscle contraction (GO:0006940)	0.000002241	0.002224	13.54
single-organism behavior (GO:0044708)	0.00001216	0.005138	12.57

GO Molecular Function 2015

Name	P-value	Adjusted p-value	Combined score
active transmembrane transporter activity (GO:0022804)	8.582e-7	0.0005710	18.97
secondary active transmembrane transporter activity (GO:0015291)	0.000001289	0.0005710	17.91
cation channel activity (GO:0005261)	0.000004843	0.0007151	17.58
symporter activity (GO:0015293)	0.000001992	0.0005882	17.11
ligand-gated ion channel activity (GO:0015276)	0.000004454	0.0007151	16.59
ligand-gated channel activity (GO:0022834)	0.000004454	0.0007151	16.50
metal ion transmembrane transporter activity (GO:0046873)	0.00001624	0.001898	15.54
dicarboxylic acid transmembrane transporter activity (GO:0005310)	0.00007557	0.003980	14.87
carboxylic acid transmembrane transporter activity (GO:0046943)	0.00001714	0.001898	14.45
organic acid:sodium symporter activity (GO:0005343)	0.00003719	0.002995	14.20
